# Nursing communication handover in emergency department

**DOI:** 10.1080/07853890.2021.1896174

**Published:** 2021-09-28

**Authors:** Cidália Castro, Maria do Céu Marques, Célia Tavares Vaz

**Affiliations:** a Nurse Department, Escola Superior de Saúde Egas Moniz (ESSEM), Egas Moniz Cooperativa de Ensino Superior, Caparica, Portugal; b Centro de Investigação Interdisciplinar Egas Moniz (CiiEM), Egas Moniz Cooperativa de Ensino Superior, Caparica, Portugal; c Unidade de Investigação e Desenvolvimento em Enfermagem (UI&DE), Lisboa, Portugal; d Mestranda em Enfermagem médico-cirúrgica, vertente: A pessoa em Situação crítica (IPS, UE, ESSP, IPCB, IPB), Setúbal, Portugal; e Escola Superior de Enfermagem S. João de Deus, Universidade de Évora, Évora, Portugal; f Nurse specialist in Medical-Surgical Nursing in Centro Hospitalar Barreiro Montijo EPE, Barreiro, Portugal

## Abstract

**Introduction:**

Effective communication in the transition of care is fundamental to improve patient safety and contribute to the reduction of adverse events [1]. A study carried out in 2014, in fifty-five hospital units in Portugal, under the “Evaluation of Patient Safety Culture in Hospitals”, concluded that patient safety culture is not yet widely acknowledged as a priority for health professionals [2], and that 70% of adverse health events occur due to communication failures among health professionals during the transition of care. Ineffective communication can be found in different health contexts, being more frequent during the transition of care, when it is essential to manage situations quickly and effectively. The peri-operative period, the ICU and emergency department are examples of contexts where communication processes are complex and prone to errors.

**Objective:**

To know nurses opinion about the transition of care in the emergency department, as well as their knowledge on the patient safety.

**Materials and Methods:**

This is a descriptive and exploratory study with a quantitative approach. Non-probabilistic and convenience sample. This study intends to answer the following research questions: What is the opinion of the emergency department nurses about the time of transition of care during shift change? Do nurses know the guidelines for patient safety in the care transition? A questionnaire was used as a data collection instrument. It consists of three parts: a first part on sample characterisation; a second part that seeks to know the opinion of nurses about the transition of care in the change of shift; and a third part, with the objective of assessing nurses' knowledge on patient's safety. The questionnaire was applied during the month of January 2019.

**Results:**

Of the total of seventy questionnaires delivered fifty were returned, with a response rate of 67.57%. The sample is essentially composed of women (82%), with a mean age of 33.46 years. They have on average 10.67 years as nurses and 7.29 years as nurses in the emergency department. With regard to nurses' opinion on the transition of care during shift change, four domains were found, namely: Positive aspects of the nursing care transition moment; Negative aspects of the nursing care transition moment; Patient evaluation at the moment of nursing care transition; and management of the information obtained during the nursing care transition. Regarding nurses' knowledge on patient safety, three areas were identified: Knowledge of the guidelines on effective communication in the transition of care; Benefits of using a standard tool in the transition of care, and training in the area of patient safety. **Discussion and conclusions:** Nurses feel that there are a number of factors that interfere with the transition of care; there is irrelevant information that is transmitted in the moment of transition of care and the ISBAR methodology contributes to decision-making and critical thinking. It is important to promote team training in the area of patient safety. Nurses have the legal obligation to ensure continuity of care through effective communication, using existing resources, namely the ISBAR tool.
